# Inverse Design of Low-Resistivity Ternary Gold Alloys via Interpretable Machine Learning and Proactive Search Progress

**DOI:** 10.3390/ma17143614

**Published:** 2024-07-22

**Authors:** Hang Che, Tian Lu, Shumin Cai, Minjie Li, Wencong Lu

**Affiliations:** 1 Department of Chemistry, College of Sciences, Shanghai University, Shanghai 200444, China; chehang@shu.edu.cn (H.C.); caishumin@shu.edu.cn (S.C.); 2 Shanghai Shuzhiwei Information Technology Co., Ltd., 668 ShangDa Road, Shanghai 200444, China; luktian05@gmail.com

**Keywords:** material discovery, machine learning, inverse design, TGAs, ρ

## Abstract

Ternary gold alloys (TGAs) are highly regarded for their excellent electrical properties. Electrical resistivity is a crucial indicator for evaluating the electrical performance of TGAs. To explore new promising TGAs with lower resistivity, we developed a reverse design approach integrating machine learning techniques and proactive searching progress (PSP) method. Compared with other models, the support vector regression (SVR) was determined to be the most optimal model for resistivity prediction. The training and test sets yielded R^2^ values of 0.73 and 0.77, respectively. The model interpretation indicated that lower electrical resistivity was associated with the following conditions: a van der Waals Radius (*V*_rt_) of 0, a *V*_r_ (another van der Waals Radius) of less than 217, and a mass attenuation coefficient of MoKα (*M*_acm_) greater than 77.5 cm^2^g^−1^. Applying the PSP method, we successfully identified eight candidates whose resistivity was lower than that of the sample with the lowest resistivity in the dataset by more than 53–60%, e.g., Au_1.000_Cu_4.406_Pt_1.833_ and Au_1.000_Pt_2.232_In_1.502_. Finally, the candidates were validated to possess low resistivity through the pattern recognition method.

## 1. Introduction

The application of ternary gold alloys (TGAs) spans across a diverse range of applications, encompassing microelectronic devices, electrode materials, and electrocatalytic reactions [1,2,3]. In sectors related to electricity, electrical resistivity stands out as a critical performance metric. Usually, a lower electrical resistivity contributes to improved electricity transmission efficiency, reduced energy losses, decreased heat generation, and the mitigation of issues such as signal interference [4,5]. Historically, the identification of new alloy compositions has been a complex process, often relying on conventional trial-and-error experiments. However, in the case of alloys containing precious metals like TGAs, trial-and-error experiments become cost-prohibitive and impractical, possibly accounting for the scarcity of publications on this subject.

With the ongoing evolution of computer technology, a multitude of pertinent material design techniques have emerged, such as quantum chemical computations, molecular dynamics simulations [6], density functional theory (DFT) [7,8], Monte Carlo simulations [9], etc. Currently, these methods have expanded their applications to encompass material resistivity calculations. For instance, Alfè et al. used collinear spin-polarized DFT to calculate the lattice resistivity of bcc iron [10], where the simulated estimates were found to be in accordance with the experimental data. Harukazu et al. employed DFT and Møller–Plesset second-order perturbation theory (MP2) methods to analyze the crystal structures and resistivity of three distinct TMTSF (tetramethyltetraselenafulvalene) salts [11]. They proposed a computational method for determining the non-integer valence of TMTSF molecules within crystalline structures. The results of their work indicated that this method provided valence states of TMTSF molecules in I_3_^−^ salts consistent with their electrical properties. Zhang et al. used first-principles molecular dynamics (FPMD) and dynamical mean field theory (DMFT) to calculate the resistivity and thermal conductivity of Fe-Si alloys [12]. Raghuraman et al. used DFT to calculate the resistivity of high-entropy alloys [13].

While DFT calculations have proven effective in specific systems [14,15,16], their applications in alloy systems, which involve combinations of multiple elements, significantly increases computational complexity, demanding substantial computational resources and time. Machine learning (ML) has demonstrated itself as a simple and efficient method that has been successfully applied in the design of alloy materials. For example, Roy et al. employed a Generative Adversarial Network (GAN) model to design high-hardness Multi-Principal Element Alloys (MPEAs) [17]. Through a search in an 18-element space, one of the alloys designed exhibited a hardness (941HV) 10% higher than the training data (857HV). Deffrennes et al. [18]. developed a framework for predicting binary liquidus, utilizing data from 466 CALPHAD binary phase diagrams to establish three machine learning models for predicting the formation of liquid miscibility gaps, the equilibrium onset temperature of solidification, and the liquid miscibility gap temperature. Ma et al. utilized a combination of the Non-dominated Sorting Genetic Algorithm II (NSGA-II) and virtual screening to optimize the composition of high-entropy alloys [19]. They ultimately recommended three candidate samples, all of which showed a significant improvement in Vickers’ hardness and compressive fracture strain compared to the original dataset. Feng et al. designed low-content Al-Mg-Si alloys that exhibited mechanical properties comparable to the other series aluminum alloys [20]. It is worth noting that low-Cu content Al alloys tended to have better corrosion resistance. The successful application of ML techniques in these studies demonstrates their significant potential in alloy research. Although TGAs have been identified as suitable materials for various applications, the number of relevant studies on TGAs is extremely limited [3]. Recently, we used the SVR model to predict the electrical resistivity of TGAs and employed the high-throughput screening approach to design a series of candidate samples [21]. However, constrained by computational power and the impracticality of exhaustive enumeration, only a small fraction of the TGA chemical space has been explored. Therefore, more effort is still required to conduct more meaningful research into TGAs.

In this work, we introduced an inverse design framework that integrated an interpretable ML model and proactive searching progress (PSP) to design low-resistivity TGAs. First, 51 samples were collected from the literature to construct the dataset. Subsequently, feature engineering was conducted, including descriptor imputation, data preprocessing, and feature selection. After comparing a series of models, the support vector regression (SVR) model was selected for modeling and SHapley Additive exPlanations (SHAP) was used for model interpretation [22,23]. The self-developed PSP method was then employed to design new TGAs with low resistivity, and the candidates were validated using a pattern recognition method. This inverse design framework will provide significant guidance for discovering low-resistivity TGAs and can be applied to the design of other materials.

## 2. Method

**Figure 1:**
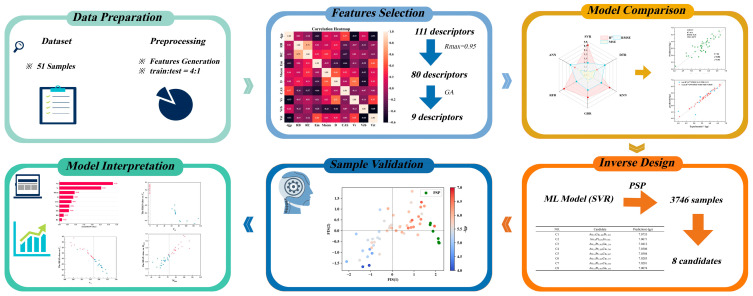
Workflow of machine learning.

The framework of the inverse design of the low-resistivity TGAs is depicted in Figure 1. The dataset was composed of 51 TGA samples paired with 111 generated atomic descriptors for our ML approach. The Pearson correlation and genetic algorithm (GA) [24] were sequentially used for the feature selection to determine the optimal feature set. By comparing various model algorithms, a well-fitted SVR model was established, and the model interpretation was accessed via SHAP method. The reverse design was carried out by integrating the SVR model with the PSP method to identify the new promising TGAs. The search objective was set to identify the TGAs whose negative logarithm of resistivity −lgρ exceeded 8 to go beyond the highest value of 6.68 in the current samples as much as possible. Regarding the newly explored TGAs, pattern recognition was used for sample validation, where the result proved the new samples have potential to have lower resistivity.

### 2.1. Dataset

The 51 TGA samples were collected from the studies accessible from the Materials Platform for Data Science (MPDS), with the majority featured in our previous work [21]. Among these 51 samples, there are 15 different elements, with Au as element A, and 15 elements used for elements B and C. A total of 111 descriptors, filled using the Villars and Mendeleev databases, were used for the subsequent work.

### 2.2. Model Construction

Feature selection is very important and can improve model performance and reduce computational costs [25]. Concerning the 111 descriptors, Pearson correlation was firstly used to prune the highly correlated features, resulting in 80 descriptors. Sequentially, the GA method was used to determine the best feature subset from the remaining features. Various ML algorithms were considered and compared for the optimal model, including SVR, decision tree regression (DTR) [26], K-nearest neighbors (KNN) [27], random forest regression (RFR) [28], and gradient boosting regression (GBR) [29].

### 2.3. Evaluation Metrics

Leave-one-out cross-validation (LOOCV) is particularly suitable for studies with small datasets as it considers more possibilities for dividing the training and test sets, thus reducing bias from random divisions. This work is a typical example of small data machine learning, and using LOOCV effectively prevents model overfitting or underfitting caused by significant differences in dataset splits. In pursuit of a reasonable model, LOOCV was used on the training set to check the model’s robustness and fitness, and the test set was evaluated to assess the generalization ability. Additionally, the dataset was re-partitioned randomly 50 times for rebuilding the models to evaluate the model’s stability.

The common metrics, coefficient of determination (R^2^) and root mean square error (RMSE) were used to indicate the performance of the model. R^2^ can be used to measure the correlation between predictions and observations, and the value ranges from −1 to 1. The calculation formula could be depicted as follows:

(1) $$R^{2}=1-\frac{\sum_{i}({\hat{y}}_{i}-y_{i}{)}^{2}}{\sum_{i}({\bar{y}}_{i}-y_{i}{)}^{2}}$$

where $\bar{y}$ is the mean value of the observed values *y*, $y_{i}$ is the observed value, and ${\hat{y}}_{i}$ is the predicted value. RMSE can be used to measure the general error of the model. A smaller RMSE indicates a more accurate predicting ability of the model. The calculation formula can be written as follows:

(2) $$RMSE=\sqrt{\frac{1}{n}\sum_{i=1}^{n}\left(y_{i}-{\hat{y}}_{i}\right)}$$

where n is the number of samples, $y_{i}$ is the true value, and ${\hat{y}}_{i}$ is the predicted value.

### 2.4. PSP

A well-established ML model could help us access the mapping association between the chemical compositions and target resistivity, which might facilitate the design of new materials. However, as for the terms of TGAs, in addition to Au, there are 2 combinations of elements that need to be considered from the 15 elements (excluding Au) included in the entire dataset. Thus, there are $C_{15}^{1}\times C_{14}^{1}$ different elemental combinations for TGAs, concerning 15 choices for B and C sites. Given the mole fraction step of 0.01, the chemical space for TGAs could be composed of $C_{15}^{1}\times C_{14}^{1}\times 10^{6}$ different compositions, which is thousands of times larger than the one in our previous work. It may take unaffordable times to obtain all the model predictions, even for the case of quaternary element alloys.

In this study, the PSP method, proposed in our previous works [30], was employed to efficiently design new low-resistivity TGAs, rather than the traditional high-throughput screening method. The loss function of PSP was defined as the absolute error between the expected property and the property predicted by a high-accuracy model, i.e., $EE=\left|o-o_{*}\right|$, where EE is the Expected Error. The core idea of the PSP method is to consider the composition as the parameters awaiting optimization, and adopt a computationally economical surrogate model, e.g., Gaussian process regression (GPR) [31], to replace the SVR model locally. Throughout the iterative process, it allows for the rapid and efficient identification of TGAs that meet or closely match our predetermined resistivity criteria. Moreover, this inverse design method has already been successfully applied to materials design [32,33]. Thence, the PSP method could be used to determine the potential low-resistivity TGAs directly without going through all the possible predictions.

### 2.5. Pattern Recognition Validation

Pattern recognition is the process of utilizing computer algorithms to identify patterns and regularities within data. Its applications span a wide range of fields, including image recognition, data analysis, structural health monitoring, and materials science, among others. There are various methods within pattern recognition, such as principal component analysis (PCA) [34,35], partial least squares (PLS) [36], Fisher projection [37], and many more. In this study, we used pattern recognition to analyze the 2-dimensional tendency of the TGA samples and determine whether the designed samples were localized around the samples with low resistivity.

## 3. Results and Discussions

### 3.1. Data Preparation

**Figure 2:**
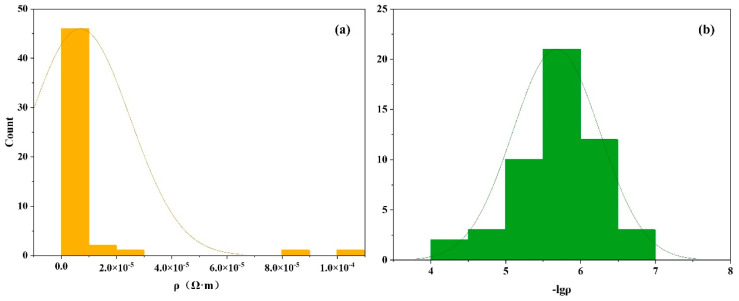
Frequency distribution histograms of (**a**) the distribution of the original dataset and (**b**) the distribution of the dataset after taking the negative logarithm.

In our study, the dataset was composed of 51 TGA samples with experimental resistivity measurements [21]. As shown in Figure 2a, the resistivity distribution of the majority of samples in the dataset is centered around $1.0\times 10^{-5}\;\Omega \cdot m$. A logarithmic transformation with a base of 10 was applied to the resistivity values to make the dataset more closely resemble a normal distribution, aiming to enhance the model’s performance [38], where the transformed distribution was plotted in Figure 2b, exhibiting a closely approximate normal distribution. The average value of −lgρ is 5.68, with a standard deviation of 0.58. Following this preprocessing step, the dataset was partitioned into a 4:1 ratio, designating 80% for training purposes and reserving 20% for an independent test set. This partitioning scheme facilitated the subsequent modeling and evaluation of the model’s generalization capabilities.

### 3.2. Feature Selection

We populated the dataset with 111 atomic descriptors sourced from the Villars and Mendeleev databases, and the descriptor details can be seen in Table S1. The features were standardized using the standardization method from the ‘preprocess’ module in scikit-learn to avoid the effects of different scales. Feature selection plays a pivotal role in ML processes, as the judicious choice of features forms the foundation of model accuracy. To mitigate the risk of dimensionality issues and overfitting, it is essential to reduce the feature numbers as much as possible.

**Figure 3:**
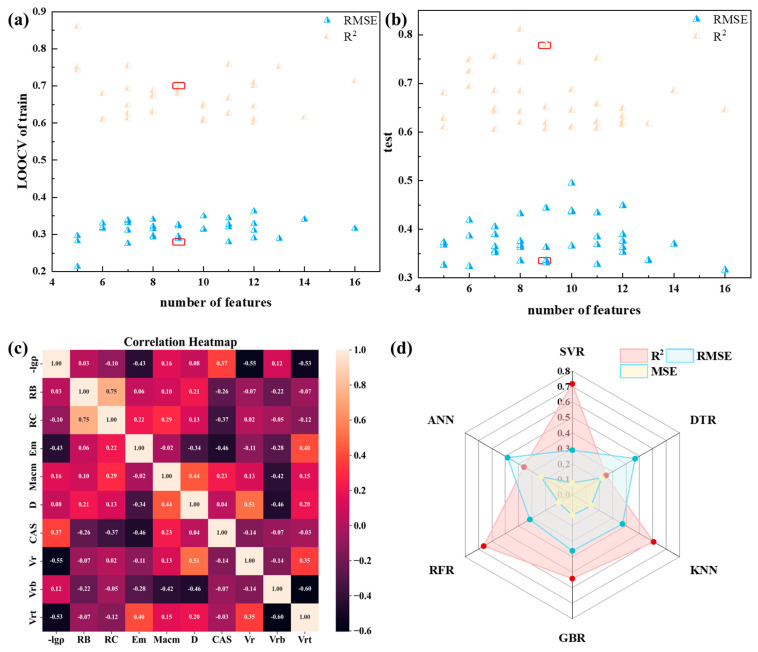
Feature selection and model comparation. (**a**) The R^2^ and RMSE of each feature subset in the training set. (**b**) The R^2^ and RMSE of each feature subset in the test set. (**c**) Correlation of 9 features. (**d**) The RMSE and R^2^ values for each algorithm on the training set using LOOCV. Note that the red frames represent selected features.

This study employed a two-step approach for feature selection. Firstly, a preprocessing step was conducted by setting the Pearson correlation coefficient threshold between features to 0.95, resulting in the retention of 80 features. Subsequently, GA was utilized for feature selection. It is worth noting that GA algorithms are inherently embedded and require integration with corresponding algorithms. We comprehensively evaluated the LOOCV results of the training set and test set, as shown in Figure 3a,b, where Figure 3a represents the results of the GA combined with the SVR algorithm (as the example). The features within the red box exhibited good performance in both the training set and test set. Consequently, we ultimately selected these nine features for further analysis. The correlation among these nine features is illustrated in the accompanying Figure 3c, demonstrating the absence of strong inter-feature correlations. The specific meanings represented by these nine features are as shown in Table 1.

### 3.3. Model Construction and Evaluation

As seen in Figure 3d, besides SVR model, we also explored the other range of algorithms including DTR, KNN, RFR and GBR to compare the performance of various models, by using the same feature selection as described above. As a result, the SVR model performed the best, achieving the highest LOOCV R^2^ value of 0.72 and the lowest RMSE of 0.288 on the training dataset. We also performed an analysis of variance (ANOVA) on the absolute values of the residuals from the LOOCV of the aforementioned algorithms and conducted post hoc analysis using the Tukey HSD test. Details can be found in the supplementary information. The ANOVA and Tukey HSD results indicate that there is no significant difference in prediction among the algorithms used in our study. However, since the SVR model showed relatively better prediction metrics, we proceeded with the SVR algorithm for the subsequent modeling. Additionally, we considered that the inclusion of the *CAS* feature in the model might not have significant physical meaning, so we contemplated removing it. After removing the *CAS* and using the remaining eight descriptors, the R^2^ value dropped to 0.464, the R value decreased to 0.736, the RMSE became 0.399, and the MSE became 0.159. This indicates that the model performance declined after removing the *CAS* feature. Therefore, we decided to retain the *CAS* feature. The specific meanings and methods of feature generation related to *CAS* can be found in the Supplementary Materials.

**Figure 4:**
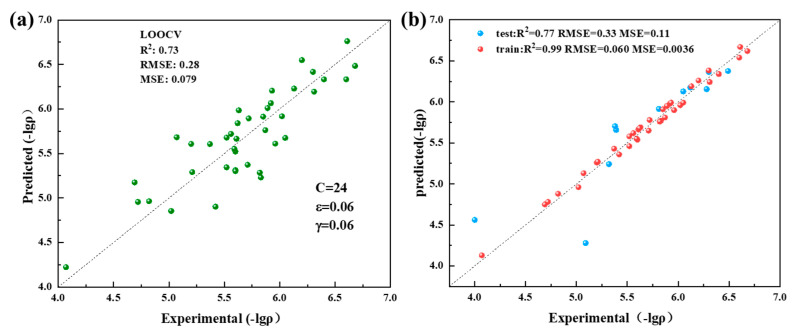
Model performance after parameter optimization. (**a**) The LOOCV results of GA-SVR using the optimized parameters on the training dataset. (**b**) Predicted values and actual values on the independent test set and training set.

Once the model was selected, fine-tuning its parameters became pivotal for enhancing modeling performance. In the case of the SVR model, this study employed a grid search method for parameter optimization. With these optimized parameters, the R^2^ value reached 0.73 during LOOCV (Figure 4a), confirming the validity of our parameter choices.

Model generalization refers to how well the model performs on data outside the training set. In this study, we assessed the model’s generalization ability using an independent test dataset (Figure 4b). The R^2^ and RMSE values on the testing dataset were 0.77 and 0.332, respectively, which are close to the results on the training dataset. This confirms that the model possesses satisfactory generalization capability.

To assess the stability of our model, we randomly divided the dataset into training and test sets in a 4:1 ratio and repeated this process 50 times for modeling. The results of the model stability test are shown in Table 2, and more details can be found in Table S2 of the Supplementary Materials. The average R² value of LOOCV was 0.68, with an average R of 0.84 and an average RMSE of 0.308. The average R² value on the test set was 0.69, with an average R of 0.87 and an average RMSE of 0.345. These findings demonstrated that the model exhibits good stability.

### 3.4. Model Interpretation

**Figure 5:**
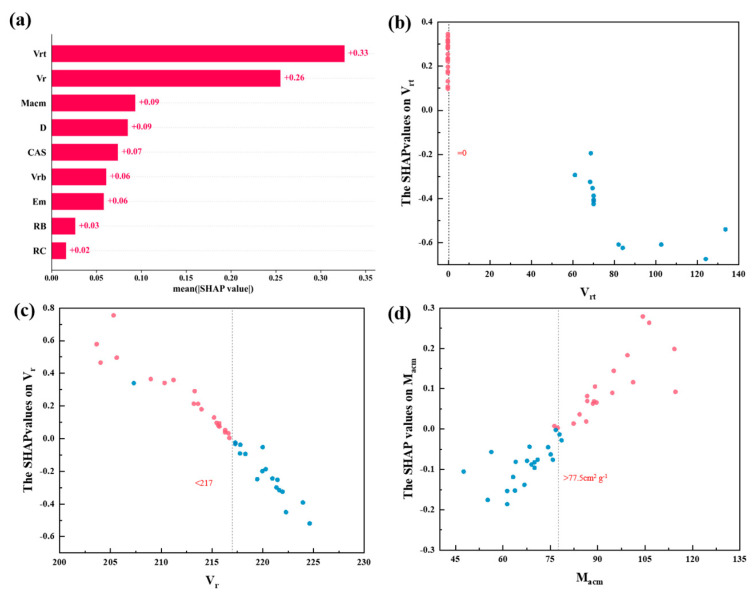
Explaining models using SHAP. (**a**) Feature importance ranking, (**b**) SHAP values of *V_rt_*, (**c**) SHAP values of *V_r_*, and (**d**) SHAP values of *M_acm_*. Note that red is positive and blue is negative.

Model interpretation can help us better understand the model. SHAP was employed to interpret the model in this study. Figure 5a displays the importance of the ranked descriptors. In the depicted graph, the feature importance is arranged in descending order as follows: *V*_rt_, *V*_r_, *M*_acm_, and other features. To uncover more valuable patterns, we partitioned the SHAP values of the most important features into positive and negative regions, as depicted in Figure 5b–d. The color of the scatter plot, whether blue or red, represents the level of influence, which illustrates the main correlations between each feature of the SVR model and the target value.

*V*_rt_ refers to the van der Waals Radius calculated by Mantina et al. [41]. As shown in Figure 5b, when *V*_rt_ equaled 0, the corresponding SHAP values were positive, and when it exceeded 0, the SHAP values became negative, indicating a negative correlation between *V*_rt_ and SHAP values. In other words, *V*_rt_ was negatively correlated with the electrical resistivity of TGAs, and when it equaled 0, the samples tended to have lower ρ. In our dataset, samples with lower electrical resistivity, such as AuCu_4_Lu, AuCu_0.25_V_0.013_, and AuLu_0.5_In_0.5_, had electrical resistivity values of $2.09\times 10^{-7}\;\Omega \cdot m$, $2.45\times 10^{-7}\;\Omega \cdot m$, and $4.09\times 10^{-7}\;\Omega \cdot m$, and their *V*_rt_ values were all equal to 0.

*V*_r_ represented another kind of van der Waals Radius defined by Bondi et al. [39] and Mantina et al. [41]. When *V*_r_ was less than 217, the corresponding SHAP values were primarily positive, and when it exceeded 217, the SHAP values became negative, indicating a negative correlation between *V*_r_ and SHAP values. In other words, *V*_r_ was negatively correlated with electrical resistivity. For example, in the dataset, the corresponding electrical resistivity values for AuCu_4.0_Lu, AuCu_0.25_V_0.013_, and AuNd_0.50_Ge are $2.09\times 10^{-7}\;\Omega \cdot m$, $2.45\times 10^{-7}\;\Omega \cdot m$, and $2.04\times 10^{-5}\;\Omega \cdot m$, while their corresponding *V*_r_ values are 203.67, 210.37, and 217.8, respectively.

*M*_acm_ stands for the mass attenuation coefficient of MoKα. As shown in Figure 5c, when *M*_acm_ exceeded a certain value of 77.5 cm^2^g^−1^, SHAP values were positive, indicating a positive correlation between *M*_acm_ and SHAP values. When *M*_acm_ exceeded 77.5 cm^2^g^−1^, the electrical resistivity of TGAs tended to be lower. For AuLu_0.5_In_0.5_, AuY_0.5_In_0.5_, and AuCeGe, the corresponding electrical resistivity values are $2.51\times 10^{-7}\Omega \cdot m$, $2.98\times 10^{-7}\Omega \cdot m$, and $1.91\times 10^{-5}\Omega \cdot m$, with their respective *M*_acm_ values being 86.88, 89.83, and 76.00.

Through the analysis mentioned above, we can conclude that when *V*_rt_ = 0, *V*_rt_ < 217, and *M*_acm_ > 77.5 cm^2^g^−1^, the −lgρ of TGAs tended to be lower. Additionally, the research of Zefirov and Zorkii also indicates that van der Waals radii influence the packing between crystals [42], which significantly impacts their electrical conductivity.

### 3.5. Model Application

#### 3.5.1. Proactive Search Process

In pursuit of the exploration and design of TGAs characterized by low resistivity, we employed the self-developed PSP method as outlined in reference [30]. High-throughput screening typically involves generating a large number of virtual samples for prediction and then selecting samples that meet the desired criteria based on the predicted target values. PSP leverages a meticulously trained model to pinpoint the samples within the designated compositional space that closely approximate the predefined target values. Notably, this approach, in contrast to forward design methodologies, offers substantial time savings and enhanced efficacy in uncovering samples that align with the specified criteria.

The compositional space involved linking site A with Au in a fixed proportion of 1, while sites B and C could host elements other than Au selected from the dataset, with proportions varying from 0 to 7. Given that the maximal value of −lgρ within the dataset stood at 6.68, we set the −lgρ target threshold as 8, signifying the pursuit of samples characterized by the lowest feasible resistivity. Through meticulous active searching, we identified a series of virtual candidates whose −lgρ exceeded 7. Among these candidates, we curated a selection of eight instances (Table 3 that featured elements not in concurrence with one another, thereby earmarking them for prospective experimental reference. As seen in Table 3, the −lgρ of these eught candidates was lower than that of the originals, whose −lgρ ranged from 7.0074 to 7.0733. Accordingly, the ρ range was $8.45\times 10^{-8}$–$9.83\times 10^{-8}$, approximately 53–60% less than the lowest ρ $20.9\times 10^{-8}$ (−lgρ = 6.68) from the dataset, and also 18–30% lower than the ρ $12.7\times 10^{-8}$ in our previous work.

#### 3.5.2. Pattern Recognition

In this study, we employed the Fisher projection method to project the samples from high-dimensional space into two-dimensional space, where the sample projections were plotted by FIS(1) and FIS(2) derived from the linear combinations of the original features:

$$FIS\left(1\right)=-0.05617R_{B}+0.05980R_{C}-0.01009E_{m}+0.003075M_{acm}+2.098D+0.01701CAS-0.08600V_{r}-0.002259V_{rb}-0.01346V_{rt}+12.415\;$$

$$FIS(2)=+0.4580R_{B}-0.1134R_{C}-0.09091E_{m}+0.02982M_{acm}-1.517D-0.0002584CAS-0.01656V_{r}+0.002266V_{rb}-0.004873V_{rt}+6.050$$

**Figure 6:**
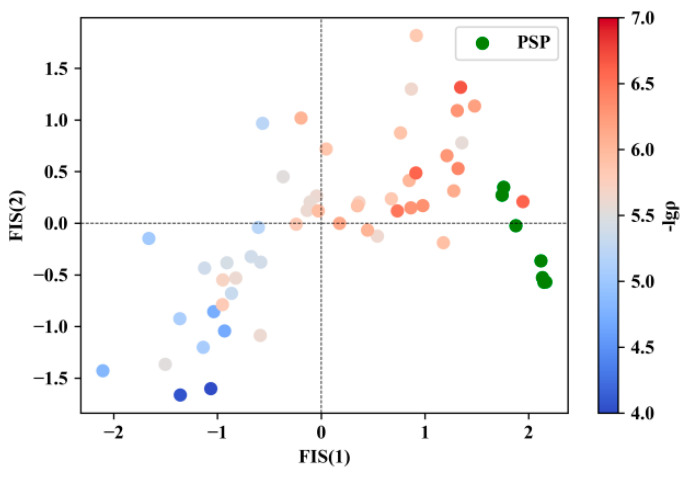
Scatter plot for pattern recognition, where red represents positive samples, blue represents negative samples, and green represents designed samples.

As seen in Figure 6, the red and blue colors represent higher and lower −lgρ values, respectively. A distinct distribution can be observed: the red samples are almost located at the top right corner of the 2D scatter plot, while the blue samples are localized around the bottom left corner, indicating the locations of the TGAs with higher and lower −lgρ values. The candidate samples designed (highlighted in green) based on the PSP results are located on the right side of the plot, closer to the TGA samples with higher −lgρ values. This discovery further validated the feasibility of PSP in designing ternary gold alloys with low electrical resistivity.

#### 3.5.3. Data Visualization

Data visualization is the representation of data in the form of images, charts, graphs, or other visual formats, making it easier to comprehend trends and patterns within the data [43,44]. In this study, we employed data visualization to better understand the trends and patterns in the electrical resistivity of the samples generated through PSP.

**Figure 7:**
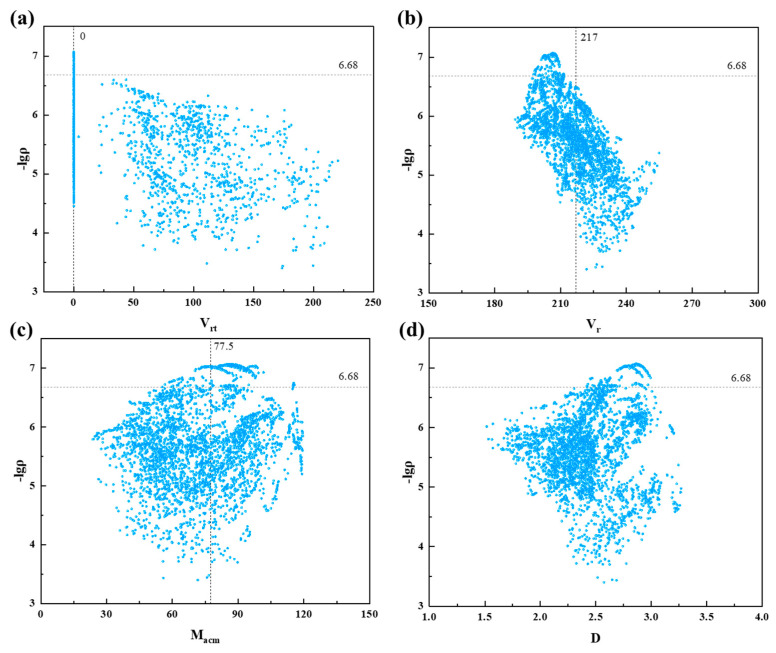
Visualization of descriptors: (**a**) *V_rt_*, (**b**) *V_r_*, (**c**) *M_acm_*, and (**d**) *D*. Note that the horizontal dashed line represents the minimum resistivity value in the dataset (−lgρ = 6.68).

As shown in Figure 7, regarding the generated PSP samples, we could observe certain trends: samples with *V*_rt_ equal to 0 tended to have lower resistivity rates, there was a higher concentration of samples with lower resistivity around *V*_r_ near 217, and there was an increased presence of samples with lower resistivity when *M*_acm_ equaled 77.5. These patterns aligned with the findings in Section 3.4 of our study.

Furthermore, we noticed that among the PSP-generated samples, there was a higher number of samples surpassing the lowest resistivity observed in the original dataset (sample details can be found in the supplementary information). This illustrated the superiority of the PSP approach in reverse materials design compared to traditional virtual screening methods.

### 3.6. Comparation of Related Work

Previously, a study by Wang et al. also employed machine learning methods [21], using virtual screening to design low-resistivity TGA materials. In their research, they designed 10 candidate materials, among which the one with the lowest resistivity was Au_0.95_Lu_0.01_In_0.04_, with a resistivity of $1.20\times 10^{-7}\;\Omega m$, −lgρ = 6.9217. In our study, we used the PSP method, which not only eliminated the need to exhaustively search the material space, but also resulted in a candidate material (Au_1.000_Cu_4.406_Pt_1.833_) with a minimum resistivity of $8.45\times 10^{-8}\;\Omega m$, nearly 30% lower than that of Au_0.95_Lu_0.01_In_0.04_. Furthermore, compared to the candidate materials proposed by them (Au_x_Lu_y_In_z_), our candidate materials are more diverse in elemental composition, including Pt, In, Ga, and Cu in addition to Au. However, unfortunately, neither their work nor ours involved experimental synthesis.

## 4. Conclusions and Outlooks

In this study, we propose an inverse design framework for designing ternary gold alloys with low electrical resistivity.

(1) Based on 51 TGA samples, the SVR model was established to predict their target electrical resistance, after comparing with its counterpart models, whose LOOCV R and RMSE were 0.73 and 0.281, respectively. The independent test set also yielded an R^2^ and RMSE of 0.77 and 0.332, indicating the good fitness of the SVR model.
(2) A series of samples was designed through the utilization of our self-developed PSP method, and eight candidates were selected. The results of pattern recognition further demonstrated the feasibility of our sample design method.
(3) The SHAP method was introduced to indicate that lower electrical resistivity occurs when *V*_rt_ equals 0, *V*_r_ is less than 217, and *M*_acm_ is greater than 77.5 cm^2^g^−1^.

Our model can predict the resistivity of gold alloys with compositions different from those within the dataset, demonstrating its transferability to some extent. However, due to significant differences between materials, we are currently unable to validate the model’s transferability to other materials. Nonetheless, we will continue researching in this direction. Finally, our reverse design framework is applicable to other materials, and we encourage interested researchers to explore further with experimental validation.

## Figures and Tables

**Table 1:** The meanings and abbreviations of the 9 features used for model construction.

Features	Description
R_B_	Proportion of element B
R_C_	Proportion of element C
E_m_	Enthalpy melting (kJ mol^−1^)
M_acm_	Mass attenuation coefficient for MoKalpha (cm^2^ g^−1^)
D	Distance valence electron (Schubert) (Å)
CAS	CAS number
V_r_	van der Waals Radius [39,40,41]
V_rb_	
V_rt_	

**Table 2:** Results of the model stability test.

	LOOCV			TEST		
	R^2^	RMSE	R	R^2^	RMSE	R
average	0.68	0.31	0.84	0.69	0.34	0.87
σ	0.052	0.034	0.027	0.052	0.049	0.033

**Table 3:** Eight candidates designed using the PSP method.

NO.	Candidate	Prediction (−lgρ)
C1	Au_1.000_Cu_4.406_Pt_1.833_	7.0733
C2	Au_1.000_Pt_2.232_In_1.502_	7.0671
C3	Au_1.000_Pt_1.901_Ga_1.214_	7.0412
C4	Au_1.000_Pt_2.208_Cu_4.784_	7.0306
C5	Au_1.000_Pt_1.948_Cu_4.507_	7.0304
C6	Au_1.000_Pt_1.605_Cu_3.377_	7.0283
C7	Au_1.000_Pt_1.697_Cu_4.338_	7.0281
C8	Au_1.000_Pt_2.801_Ga_1.435_	7.0074

## Data Availability

The dataset and code are available from a GitHub link at GitHub-chehang228/PSP: data available.

## Supplementary Materials

The following supporting information can be downloaded at: https://www.mdpi.com/article/10.3390/ma17143614/s1, Table S1: 111 features filled with data from the Villars and Mendeleev databases, Table S2: Repeatedly randomly partitioning the training and test sets to validate the model’s robustness, Table S3: ANOVA results, Table S4: Tukey HSD test results and PSP samples. Reference [45] are cited in the supplementary materials.
