# Supplementary material for: Inverse Design of Low-Resistivity Ternary Gold Alloys via Interpretable Machine Learning and Proactive Search Progress
Source: Materials (Basel). 2024 Jul 22;17(14):3614. doi: 10.3390/ma17143614 (PMC11278811; doi:10.3390/ma17143614)
Supplement: Supplementary file 1 [file materials-17-03614-s001.zip › supporting information_revised.pdf]

Inverse Design of Low-Resistivity Ternary Gold Alloys via Interpretable Machine Learning and Proactive Search Progress

Hang Che[a], Tian Lu[b], Shumin Cai[c] , Minjie Li[*] and Wencong Lu[*]

Features

The meaning of the 'CAS number' is the Chemical Abstracts Service identifier. The calculation method for this feature is as follows: for example, in the dataset, AuLaIn has a CAS number of 74, where Au, La, and In have CAS numbers of '7440-57-5', '7439-91-0', and '7440-74-6', respectively. These are string types. For this type of string, we used the LabelEncoder module from scikit-learn. After encoding, '7440-57-5' was converted to 90, '7439-91-0' to 25, and '7440-74-6' to 107. After weighting, we got the feature value of 74. Similarly, there is also a feature from the Villars database called "Periodic number start counting left bottom of B-site"[1].

Table S1. 111 features filled with data from the Villars and Mendeleev databases.

Descriptor Names		
A_ratio	cas	mendeleev_h_li_na_be_mg_block_t_d_left
B_ratio	goldschmidt_class	mendeleev_h_be_mg_t_d_left
C_ratio	geochemical_class	mendeleev_h_li_na_be_mg_t_d_left
atomic_number	is_monoisotopic	distance_valence_electron
quantum_number	is_radioactive	distance_core_electron
atomic_weight	name	v2_3_miedema
group_number	atomic_number_2	atomic_env_number
valence_electron_number	atomic_number_3	covalent_radius_cordero
melting_point	atomic_number_4	covalent_radius_pyykko
boiling_point	enthalpy_surface_miedema	covalent_radius_bragg
enthalpy_vaporization	enthalpy_vacancies_miedema	covalent_radius_slater
enthalpy_melting	mass_attenuation_coef_mokalpha	covalent_radius_pyykko_double
enthalpy_atomizati	mass_attenuation_coef_cr	covalent_radius_pyykko_triple

on	kalpha	
volume_Villars	mass_attenuation_coef_crkalpha_2	vdw_radius
volume_Mendeleev	mass_attenuation_coef_fekalpha	vdw_radius_bondi
en_martynov	atomic_electron_scattering_factor	vdw_radius_truhlar
en_pauling	work_function	vdw_radius_rt
en_alfred	nws1_3_miedema	vdw_radius_batsanov
en_mulliken	nulear_charge_effective_slater	vdw_radius_dreiding
en_allen	charge_nuclear_effective_clementi	vdw_radius_uff
en_ghosh	energy_cohesive_brewer	vdw_radius_mm3
first_ionization	modulus_compression	vdw_radius_alvarez
second_ionization	modulus_bulk	atomic_radius_rahm
third_ionization	modulus_rigidity	atomic_weight_uncertainty
chemical_potential	modulus_Young	gas_basicity
radii_pseudo_zunger	mendeleev_number	heat_of_formation
radii_ionic_yagoda	mendeleev_number_2	c6
radii_metal_waber	mendeleev_number_3	c6_gb
atomic_radii	mendeleev_number_4	metallic_radius_c12
density	pettifor_number	dipole_polarizability_unc
dipole_polarizability	pettifor_number_regular	specific_heat
electron_affinity	glawe_number	fusion_heat
evaporation_heat	mendeleev_chemisits_sequence	thermal_conductivity
lattice_constant	mendeleev_t_d_left	abundance_crust
proton_affinity	mendeleev_t_d_right	abundance_sea
block	mendeleev_d_t_left	ionization
lattice_structure	mendeleev_d_t_right	_ionic_radius_

Table S2. Repeatedly randomly partitioning the training and test sets to validate the model's robustness.

NO.		LOOCV			TEST	
	R^2	RMSE	R	R^2	RMSE	R
1	0.83	0.223	0.91	0.64	0.389	0.83

2	0.82	0.222	0.91	0.73	0.380	0.86
3	0.81	0.240	0.90	0.65	0.379	0.84
4	0.78	0.279	0.90	0.60	0.302	0.87
5	0.75	0.272	0.87	0.69	0.338	0.85
6	0.74	0.297	0.88	0.70	0.309	0.86
7	0.74	0.285	0.86	0.75	0.330	0.88
8	0.72	0.275	0.85	0.62	0.399	0.80
9	0.72	0.302	0.85	0.67	0.337	0.84
10	0.71	0.311	0.86	0.61	0.336	0.90
11	0.71	0.295	0.84	0.70	0.369	0.85
12	0.71	0.270	0.85	0.62	0.421	0.79
13	0.71	0.289	0.84	0.72	0.347	0.86
14	0.70	0.304	0.84	0.72	0.337	0.86
15	0.70	0.315	0.86	0.66	0.342	0.88
16	0.70	0.311	0.85	0.72	0.333	0.88
17	0.69	0.321	0.85	0.69	0.311	0.86
18	0.69	0.315	0.85	0.74	0.316	0.87
19	0.69	0.317	0.85	0.70	0.303	0.92
20	0.69	0.296	0.83	0.77	0.342	0.89
21	0.69	0.324	0.84	0.73	0.292	0.89
22	0.68	0.280	0.83	0.77	0.370	0.88
23	0.68	0.332	0.85	0.76	0.266	0.90
24	0.67	0.308	0.82	0.67	0.397	0.86
25	0.66	0.291	0.82	0.79	0.365	0.91
26	0.66	0.340	0.84	0.70	0.299	0.86
27	0.66	0.337	0.84	0.62	0.331	0.88
28	0.66	0.336	0.83	0.73	0.305	0.87
29	0.66	0.346	0.82	0.68	0.297	0.84
30	0.65	0.303	0.81	0.78	0.355	0.90
31	0.65	0.318	0.81	0.75	0.344	0.86
32	0.65	0.337	0.83	0.73	0.301	0.86
33	0.65	0.330	0.83	0.71	0.329	0.90
34	0.65	0.267	0.82	0.76	0.401	0.87
35	0.65	0.351	0.83	0.69	0.206	0.91
36	0.65	0.343	0.85	0.67	0.331	0.89
37	0.65	0.334	0.83	0.60	0.392	0.79
38	0.65	0.333	0.82	0.72	0.334	0.89
39	0.65	0.309	0.81	0.68	0.399	0.83
40	0.64	0.319	0.83	0.73	0.367	0.86
41	0.63	0.286	0.82	0.61	0.515	0.87

42	0.63	0.348	0.82	0.69	0.311	0.91
43	0.63	0.358	0.82	0.72	0.280	0.88
44	0.63	0.338	0.82	0.69	0.358	0.89
45	0.62	0.324	0.79	0.67	0.342	0.83
46	0.62	0.360	0.81	0.67	0.283	0.85
47	0.62	0.313	0.80	0.79	0.354	0.94
48	0.62	0.354	0.81	0.69	0.326	0.83
49	0.62	0.367	0.81	0.62	0.276	0.82
50	0.62	0.372	0.81	0.60	0.285	0.80
average	0.68	0.312	0.84	0.69	0.339	0.87
σ	0.052	0.034	0.027	0.052	0.049	0.033

Table S3. ANOVA results.

Source of Variation	df	sum_sq	mean_sq	F	PR(>F)
Between Groups	5	0.612	0.123	1.856	0.103
Within Groups	234	15.453	0.066		
Total	239	16.065			

Table S4. Tukey HSD Test Results.

group1	group2	meandiff	p-adj	lower	upper	reject
residual_ANN	residual_DTR	0.0483	0.9595	-0.1168	0.2135	FALSE
residual_ANN	residual_GBR	-0.0469	0.9644	-0.212	0.1182	FALSE
residual_ANN	residual_KNN	-0.0226	0.9988	-0.1877	0.1426	FALSE
residual_ANN	residual_RFR	-0.0976	0.5341	-0.2627	0.0675	FALSE
residual_ANN	residual_SVR	-0.0892	0.6308	-0.2543	0.0759	FALSE
residual_DTR	residual_GBR	-0.0952	0.5616	-0.2603	0.0699	FALSE
residual_DTR	residual_KNN	-0.0709	0.8198	-0.236	0.0942	FALSE
residual_DTR	residual_RFR	-0.1459	0.117	-0.3111	0.0192	FALSE
residual_DTR	residual_SVR	-0.1375	0.1628	-0.3027	0.0276	FALSE
residual_GBR	residual_KNN	0.0243	0.9983	-0.1408	0.1894	FALSE
residual_GBR	residual_RFR	-0.0507	0.9504	-0.2158	0.1144	FALSE
residual_GBR	residual_SVR	-0.0423	0.9772	-0.2074	0.1228	FALSE
residual_KNN	residual_RFR	-0.075	0.7816	-0.2402	0.0901	FALSE
residual_KNN	residual_SVR	-0.0666	0.8554	-0.2318	0.0985	FALSE

Reference

1. Zhang, S.; Lu, T.; Xu, P.; Tao, Q.; Li, M.; Lu, W. Predicting the Formability of Hybrid Organic–Inorganic Perovskites via an Interpretable Machine Learning Strategy. *The*

Journal of Physical Chemistry Letters **2021**, *12* (31), 7423-7430. DOI: 10.1021/acs.jpclett.1c01939.
